# Dietary supplementation with black soldier fly (Hermetia illucens) larval meal modulates hepatic lipid homeostasis and reduces TNF-α expression in mice

**DOI:** 10.1007/s11033-026-12657-2

**Published:** 2026-08-28

**Authors:** Lucas de Oliveira Ferreira, Magno Simão Silva Filho, Katherine Costa Mello Simões, Lucyana Conceição Farias, André Luiz Sena Guimaraes, Alfredo Maurício Batista de Paula, Antônio Sérgio Barcala-Jorge, Junio Cota, João Marcus Oliveira Andrade, Sérgio Henrique Sousa Santos

**Affiliations:** 1 Food and Health Postgraduate Program, Institute of Agricultural Sciences (ICA), Federal University of Minas Gerais (UFMG), Avenida Universitária, 1000 – Universitario, 39.404-547, Montes Claros, Minas Gerais, Brazil; 2 Postgraduate Program in Health Sciences, State University of Montes Claros (UNIMONTES), Montes Claros, Minas Gerais, Brazil

**Keywords:** Edible Insects, Liver, Lipid metabolism, Nutrition, Proteins

## Abstract

**Background:**

Black soldier fly (*Hermetia illucens* L.) larval meal has attracted growing interest as a sustainable food ingredient due to its nutritional composition and potential biological effects. The present study evaluated the effects of dietary supplementation with *H. illucens* larval meal on body composition, biochemical parameters, and hepatic markers in mice.

**Methods and results:**

Twenty Swiss mice (n = 10/group) were randomly assigned to Control (AIN-93 M; mean initial body weight: 41.3 g) and AIN-93 M supplemented with 10% *H. illucens* larval meal (AIN-93 M + BSF; mean initial body weight: 39.6 g) for 12 weeks. When compared with controls, BSF-supplemented mice exhibited a 17.1% increase in body weight from baseline (final body weight: 46.36 g; p < 0.0001), whereas control mice showed a 3.5% reduction (final body weight: 39.87 g). This increase was accompanied by greater lean and fat mass (p < 0.05), while food intake and total body water remained unchanged (p > 0.05). Serum biochemical parameters, including lipid profile, ALT, and AST, showed no significant differences between groups (p > 0.05). Histological analysis revealed reduced hepatic lipid droplet area in BSF-supplemented mice (p < 0.05). Additionally, hepatic TNF-α expression was decreased (p < 0.05), whereas FASN expression was not significantly altered (p > 0.05).

**Conclusions:**

The present findings suggest that a 10% H. illucens larval meal modulates selected hepatic parameters by reducing lipid accumulation and inflammatory signaling under the conditions evaluated. Further studies are needed to clarify its long-term metabolic effects and potential applications as a sustainable dietary ingredient.

**Graphical abstract:**

**Figure:**
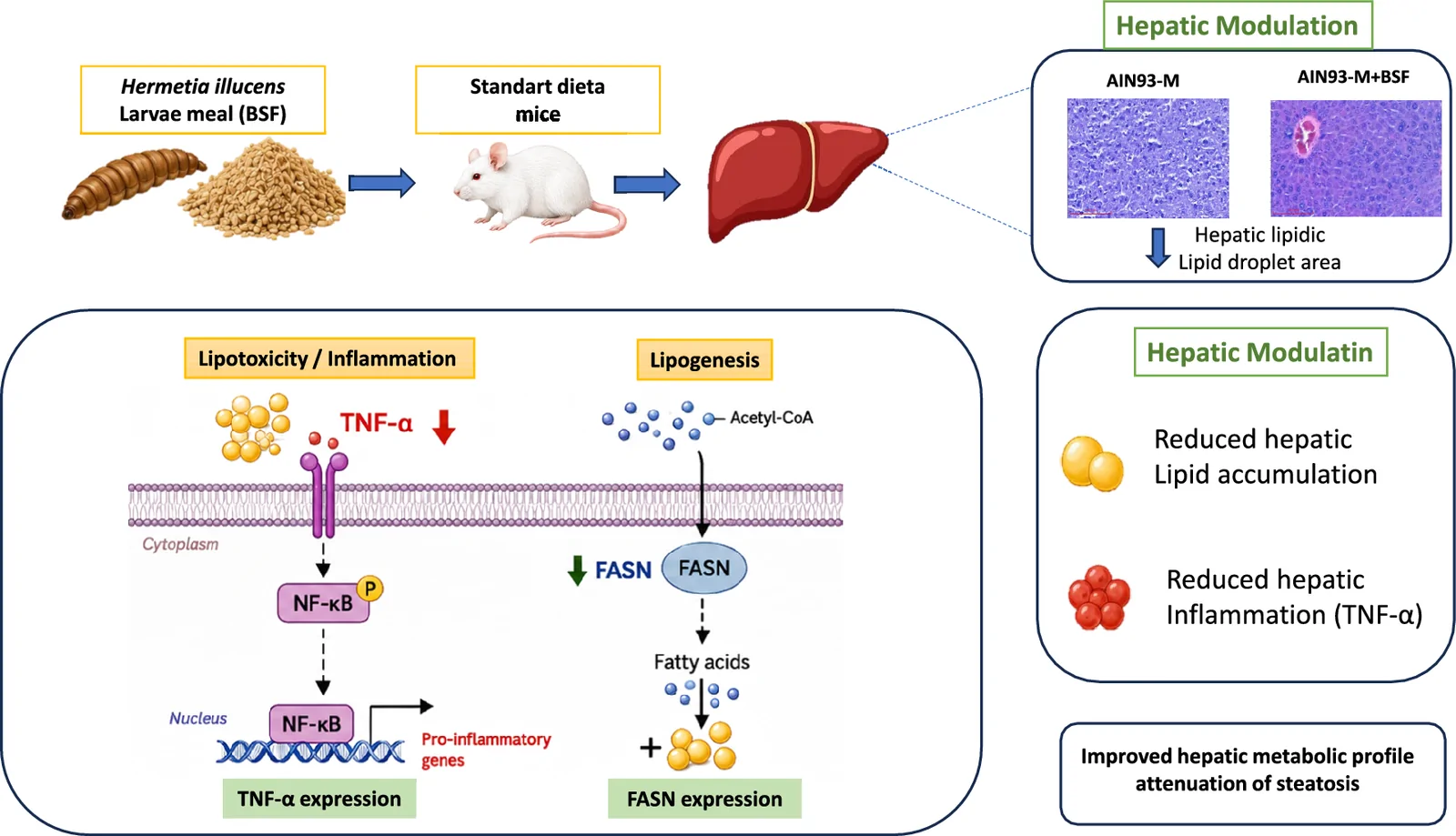

**Supplementary Information:**

The online version contains supplementary material available at https://doi.org/10.1007/s11033-026-12657-2.

## Introduction

The liver plays a central role in regulating energy metabolism, being responsible for the synthesis, oxidation, and storage of lipids, as well as maintaining glycaemic and protein homeostasis [1]. Alterations in lipid metabolic dynamics can lead to the accumulation of intracellular lipid droplets, a phenomenon associated with the development of metabolic disorders and impaired liver function [2, 3]. Consequently, even under physiological conditions, hepatic lipid synthesis and storage remain metabolically active, allowing nutritional interventions that modulate lipid homeostasis to be detected [2, 4].

In this context, the Black Soldier Fly, *Hermetia illucens* (Diptera: *Stratiomyidae*), has attracted considerable attention as a sustainable nutrient source. *H. illucens* larvae meal is notable for its high protein content (32 to 48% of dry matter, DM), high fat content (12 to 42% DM), and favorable amino acid profile, being rich in lysine, leucine, valine, isoleucine, threonine, and methionine [5, 6]. It also contains appreciable amounts of lipids, minerals, antimicrobial peptides, and chitin-derived compounds. These nutritional characteristics have supported its use in animal and human nutrition and motivated research into its potential functional properties [7]. Moreover, *H. illucens* is one of the most promising insect species for sustainable food production due to its high feed-conversion efficiency, ability to valorize organic by-products, and reduced environmental footprint compared with conventional livestock production systems. These characteristics have positioned *H. illucens* larvae among the leading candidates for future sustainable food and feed applications [8, 9].

In addition to their high nutritional value, *H. illucens* larvae are a source of several bioactive compounds. Lauric acid is the predominant fatty acid in the larval lipid fraction, accounting for approximately 36–60% of total fatty acids, followed by palmitic, oleic, myristic and linoleic acids [10]. The larvae also contain chitin- and chitosan-derived polysaccharides, as well as bioactive peptides generated from insect proteins, all of which are associated with potential health-promoting properties [11].

Lauric acid is associated with the modulation of energy and lipid metabolism and exhibits antimicrobial and anti-inflammatory properties, while chitin and its derivatives may contribute to the regulation of the intestinal microbiota and immune responses [12]. Together, these bioactive compounds may contribute to metabolic homeostasis and influence pathways involved in lipid metabolism and inflammation, suggesting a potential role in the modulation of basal metabolic responses rather than exclusively acting under pathological conditions [13, 14].

Previous studies have primarily focused on the nutritional value of *H. illucens* larvae as a sustainable protein source and on their effects on growth performance, feed efficiency, gut health, and immune responses in livestock, aquaculture species, and obesity-related experimental models [6, 15] Although these studies suggest that bioactive compounds present in the larvae may influence lipid metabolism and inflammatory pathways, evidence regarding their effects on hepatic lipid regulation under physiological conditions remains limited [16, 17]. In particular, little is known about whether dietary supplementation with *H. illucens* larvae meal can modulate hepatic lipid accumulation and inflammatory markers in healthy animals fed a standard maintenance diet. Most studies demonstrating the beneficial metabolic effects of *H. illucens* have been conducted in disease-associated models, such as obesity induced hepatic steatosis [18], or have focused on immune and antioxidant responses in other animal species [19], leaving its effects under normal physiological conditions largely unexplored. Addressing this knowledge gap is important to determine whether the functional properties of *H. illucens* extend beyond disease-associated models and contribute to the regulation of hepatic metabolism under normal physiological conditions.

Therefore, this study investigated the effects of dietary supplementation with *H. illucens* larvae meal on body composition, hepatic lipid accumulation, and the expression of genes involved in lipid metabolism and inflammation under basal physiological conditions in mice. This approach provides insights into the potential metabolic effects of *H. illucens* supplementation in healthy animals, a condition that remains little explored compared with disease models.

## Materials and methods

### Ethical approval

All procedures were carried out following the guidelines and authorization of the Ethics Committee on Animal Experimentation and Welfare of the State University of Montes Claros, under protocol 224/2021.

### Preparation of black soldier fly flour

The *H. illucens* (Black Soldier Fly, BSF) larvae used in the present study were supplied by Agrin Biotecnologia (Avaré, SP, Brazil) as previously dehydrated, vacuum-packed larvae. According to the manufacturer, the larvae were harvested at the late larval stage, before pupation, at approximately 10–12 days of age, with an average individual weight of approximately 0.5–1.0 g. Upon receipt, the material was stored in its original packaging, protected from light and moisture, until processing. The dehydrated larvae were ground in a previously sanitized blender to obtain a homogeneous flour, following the methodology described in previous studies [20]. The flour was subsequently weighed using an analytical balance and incorporated into the experimental diets at a concentration of 10% (w/w), according to the formulation established for the supplemented groups.

### Mouse model and experimental design

The study used 20 six-week-old male Swiss (*Mus musculus*) mice obtained from the Multidisciplinary Center for Biological Research in the Area of Laboratory Animal Science (CEMIB) at the State University of Campinas (UNICAMP, Campinas, SP, Brazil). At the beginning of the experimental period, the animals had similar initial body weights, with mean values of 41,3 g for the Control group and 39,6 g for the AIN-93 M + BSF group. The animals were housed in cages under controlled environmental conditions, with a 12-h light/dark cycle, a temperature of 22 ± 2 °C, and relative humidity of 60 ± 5%, with ad libitum access to water throughout the experimental period. The animals were housed in individually ventilated rack systems (Alesco, Brazil) in polycarbonate cages (41 × 34 × 18 cm). Cage maintenance was performed twice weekly using autoclaved wood shavings, and all cages were provided with Flat environmental enrichment devices (Alesco, Brazil) to minimize stress and promote animal welfare.

After an adaptation period, the animals were randomly assigned to two experimental groups (n = 10 per group): (1) the Control group, which received the standard AIN-93 M diet, and (2) the AIN-93 M + BSF group, which received the AIN-93 M diet supplemented with 10% *H. illucens* larvae meal (BSF). The experiment lasted 12 weeks (December 2024 to March 2025). The composition of the experimental diets is presented in Table 1. After the end of the treatment, the animals underwent weight measurements and tests to assess insulin sensitivity and glucose levels. Subsequently, we sacrificed the animals by decapitation, and blood, tissues, and organs of interest were collected. Tissue samples intended for molecular analyses were immediately frozen and stored at −80 °C, whereas samples for histological evaluation were fixed in 10% neutral buffered formalin until processing [21]. Animal carcasses were disposed of by incineration, following institutional procedures in accordance with the Brazilian guidelines established by the National Council for the Control of Animal Experimentation (CONCEA) and the Brazilian Health Regulatory Agency (ANVISA) Resolution RDC Nº. 222/2018, which regulates the management of healthcare waste, including carcasses generated from animal research facilities [22, 23].

**Table 1:** - Composition of the experimental diets AIN-93 M and AIN-93 M supplemented with Black soldier fly (BSF; *H. illucens*) larval meal

Ingredients*	AIN93-M (g/kg)	AIN93-M+BSF (g/kg)
BHT	0,01	0,01
Choline bitartrate	2,5	2,5
Methionine	1,8	1,8
Vitamin mix	10	10
Mineral mix	35	35
Cellulose	50	50
Soybean oil	40	5,83
Sucrose	100	100
Maltodextrin	155	155
Casein	140	108,24
Corn starch	465,69	445,74
BSF	—	100
Energy value (kcal/100 g)	360	360

*AIN-93 M, American Institute of Nutrition 93 maintenance diet; BSF, Black Soldier Fly (*H. illucens*) larvae meal; AIN-93 M+BSF, AIN-93 M diet supplemented with Black Soldier Fly (*H. illucens*) larvae meal; BHT, butylated hydroxytoluene

### Assessment of food intake, body weight, and body composition

Body weight (BW) and food intake were monitored daily at fixed time points using semi-analytical balances. Individual BW was recorded in grams throughout the experimental period, and group means were calculated for each assessment day. Food intake was estimated on a per-cage basis by calculating the difference between the amount of diet offered and the remaining feed. To account for differences in body size, food intake was normalized to body weight (BW) and expressed as food intake/BW according to the following equation:

$$Food intake/BW=\frac{food intake (g/day)}{Body weight (g)}$$

This approach has been previously adopted in nutritional intervention studies evaluating food intake relative to body weight [24].

Body composition was assessed at the end of the experimental period using a Minispec LF90 Body Composition Analyzer (Bruker BioSpin, Karlsruhe, Germany), based on time-domain nuclear magnetic resonance (TD-NMR). Conscious mice were individually placed in a restraining tube specifically designed for small rodents without anesthesia. The instrument differentiates tissue-specific nuclear magnetic resonance signals to non-invasively determine fat mass, lean body mass, and total body fluid, according to the manufacturer’s instructions and as previously described by [25].

### Glucose tolerance and insulin sensitivity test

Glucose tolerance was assessed using the intraperitoneal glucose tolerance test (GTT), performed after a 6-h fast. Animals received glucose (2 g/kg body weight), and blood glucose levels were measured by caudal vein puncture at 0, 15, 30, 60, and 120 min using an Accu-Chek glucometer (Roche Diagnostics, Germany) [26].

Insulin sensitivity was assessed using the intraperitoneal insulin tolerance test (ITT), performed in fed animals. After a 1-h fast, insulin was administered at a dose of 0.75 IU/kg body weight, and blood glucose levels were determined at 0, 15, 30, and 60 min by caudal vein puncture using the same equipment [26].

### *Histological analysis*

Liver samples were collected and immediately fixed in 10% buffered formaldehyde. After fixation, tissues were progressively dehydrated through graded ethanol solutions (70–100° GL), cleared in xylene, and embedded in paraffin wax. Paraffin blocks were sectioned into 5 μm thick slices using a microtome and stained with hematoxylin and eosin (H&E) for histological evaluation.

Histological slides were digitized at 40× magnification using a Motic Easy Scan slide scanner (Motic, Xiamen, China). For morphometric analysis, five non-overlapping images per slide were randomly selected and captured at an equivalent magnification of 20x. Morphometric assessment was conducted using ImageJ software (National Institutes of Health, Bethesda, MD, USA), enabling quantification of hepatocyte areas from the acquired histological images [27].

### Reverse transcription and quantitative real-time PCR (qRT -PCR)

Total RNA was extracted from liver tissue using TRIzol™ Reagent (Invitrogen, Thermo Fisher Scientific, Waltham, MA, USA) in accordance with the manufacturer’s instructions. RNA concentration was measured using a NanoDrop™ spectrophotometer (Thermo Fisher Scientific, USA), and all samples were normalised to a final concentration of 1500 ng/µL [28, 29]. RNA integrity was then assessed using a Qubit™ 4 fluorometer and the Qubit™ RNA IQ Assay Kit (Invitrogen, Thermo Fisher Scientific, USA). Only RNA samples with RNA IQ scores indicative of acceptable integrity (≥ 7) were used for complementary DNA (cDNA) synthesis [30, 31]. RNA integrity scores for all samples are presented in Supplementary Table S1.

RNA samples were treated with DNase I to remove residual genomic DNA prior to reverse transcription. Complementary DNA (cDNA) was synthesised using M-MLV Reverse Transcriptase (Invitrogen, Thermo Fisher Scientific, USA) in accordance with the manufacturer’s instructions. Quantitative real-time PCR (qPCR) was performed using PowerUp™ SYBR™ Green Master Mix (Applied Biosystems, Thermo Fisher Scientific, USA) on a QuantStudio 6 Flex 384 well system (Applied Biosystems, Thermo Fisher Scientific, USA). All reactions were run in triplicate using gene-specific primers, which are listed in Table 2. Relative gene expression was calculated using the comparative Ct (2^−ΔΔCt^) method, with 18S rRNA as the endogenous reference gene [32].

**Table 2:** - Primer sequences, RefSeq accession numbers, and predicted amplicon sizes used for quantitative real-time PCR (qPCR) analysis of hepatic gene expression in healthy Swiss mice

*Gene	RefSeq accession	Amplicon size (bp)	Forward primer (5′–3′)	Reverse primer (5′–3′)
TNF-α	NM_013693.3	97	ATGGCCTCCCTCTCATCAGT	TTTGCTACGACGTGGGCTAC
FASN	NM_007988.3	238	CGGCTTCAGGCTCTGATACTT	CTATTCTCTACCGCTGGGGC
18S rRNA	NR_003278.3	155	AAACGGCTACCACATCCAAG	CCTCCAATGGATCCTCGTTA

*TNF-α, tumor necrosis factor alpha; FASN, fatty acid synthase; 18S rRNA, 18S ribosomal RNA. *AIN-93 M, American Institute of Nutrition 93 maintenance diet; BSF, Black Soldier Fly (*H. illucens*) larvae meal; AIN-93 M+BSF, AIN-93 M diet supplemented with Black Soldier Fly (*H. illucens*) larvae meal

### Biochemical parameters

The blood samples were centrifuged at 4000 rpm for 10 min at 4 °C to separate the serum. The resulting serum was sent to the Clemente de Faria University Hospital analysis laboratory for biochemical analyses, including total cholesterol, triglycerides, aspartate aminotransferase (AST), and alanine aminotransferase (ALT) [33].

### Determination of the centesimal composition of H. illucens larvae flour

The proximate composition of the dehydrated *H. illucens* larvae meal was determined at the Laboratory of the Institute of Agricultural Sciences, Federal University of Minas Gerais (ICA/UFMG), according to (AOAC International methods 2012) [34], as previously described for *H. illucens* larvae meal characterization. Moisture content was determined by oven-drying approximately 1.0 g of the sample at 105 °C for 24 h until a constant weight (AOAC Method 934.06). This procedure was performed exclusively for analytical moisture determination and did not represent the dehydration process used for insect meal production [20].

Crude protein content was determined by the Kjeldahl method. Approximately 0.5 g of sample was digested with 12 mL of sulfuric acid (H₂SO₄) and a catalytic mixture (1000 Kjeltabs S/3.5, Foss Tecator, Höganäs, Sweden) at 420 °C for 1 h. Total nitrogen was quantified using a Kjeltec Auto 2300 Analyzer (Foss Tecator), and crude protein content was calculated using a nitrogen-to-protein conversion factor of 4.76. The conventional factor of 6.25, commonly applied for animal proteins, may overestimate protein content in insect-derived ingredients due to the contribution of chitin-associated nitrogen; therefore, the specific conversion factor recommended for *H. illucens* was adopted [35].

Lipid content was determined by Soxhlet extraction (AOAC Method 920.85), and ash content was measured by incineration in a muffle furnace at 550 °C (AOAC Method 940.26). Available carbohydrates were calculated by difference, and the energy value was estimated using Atwater conversion factors of 4 kcal g⁻^1^ for protein, 4 kcal g⁻^1^ for carbohydrates, and 9 kcal g⁻^1^ for lipids [36].

#### Statistical analyses

Statistical analyses were performed using GraphPad Prism software, version 8.0 (GraphPad Software Inc., San Diego, CA, USA). Data are presented as mean ± standard error of the mean (SEM), and statistical significance was set at p < 0.05. Data distribution was assessed using the Shapiro–Wilk normality test. Body weight (AUC), body fat, lean body mass, body fluid, food intake, glucose tolerance test (AUC), liver weight, hepatic lipid droplet area, TNF-α expression, total cholesterol, triglycerides, aspartate aminotransferase (AST), and alanine aminotransferase (ALT) showed a normal distribution and were analysed using an unpaired Student’s t-test. In contrast, insulin tolerance test (AUC), and FASN expression did not meet the assumption of normality and were analysed using the Mann–Whitney test. The area under the curve (AUC) was calculated using the trapezoidal rule, followed by the appropriate statistical test according to the data distribution. All statistical analyses were conducted in accordance with established recommendations for experimental data analysis, Supplementary Figure S1 [37].

## Results

### Determination of the centesimal composition of H. illucens larval flour

The centesimal composition analysis showed that *H. illucens* larval meal contained a high lipid level (34.2 ± 0.62%), followed by protein (31.8 ± 0.75%) and carbohydrate (19.9 ± 0.08%). Ash and moisture contents were 8.2 ± 0.03% and 5.9 ± 0.48%, respectively. Based on this composition, the estimated energy value of the larval meal was 514.37 kcal/100 g.

### Effects of H. illucens supplementation on body weight gain and body composition

**Fig. 1:**
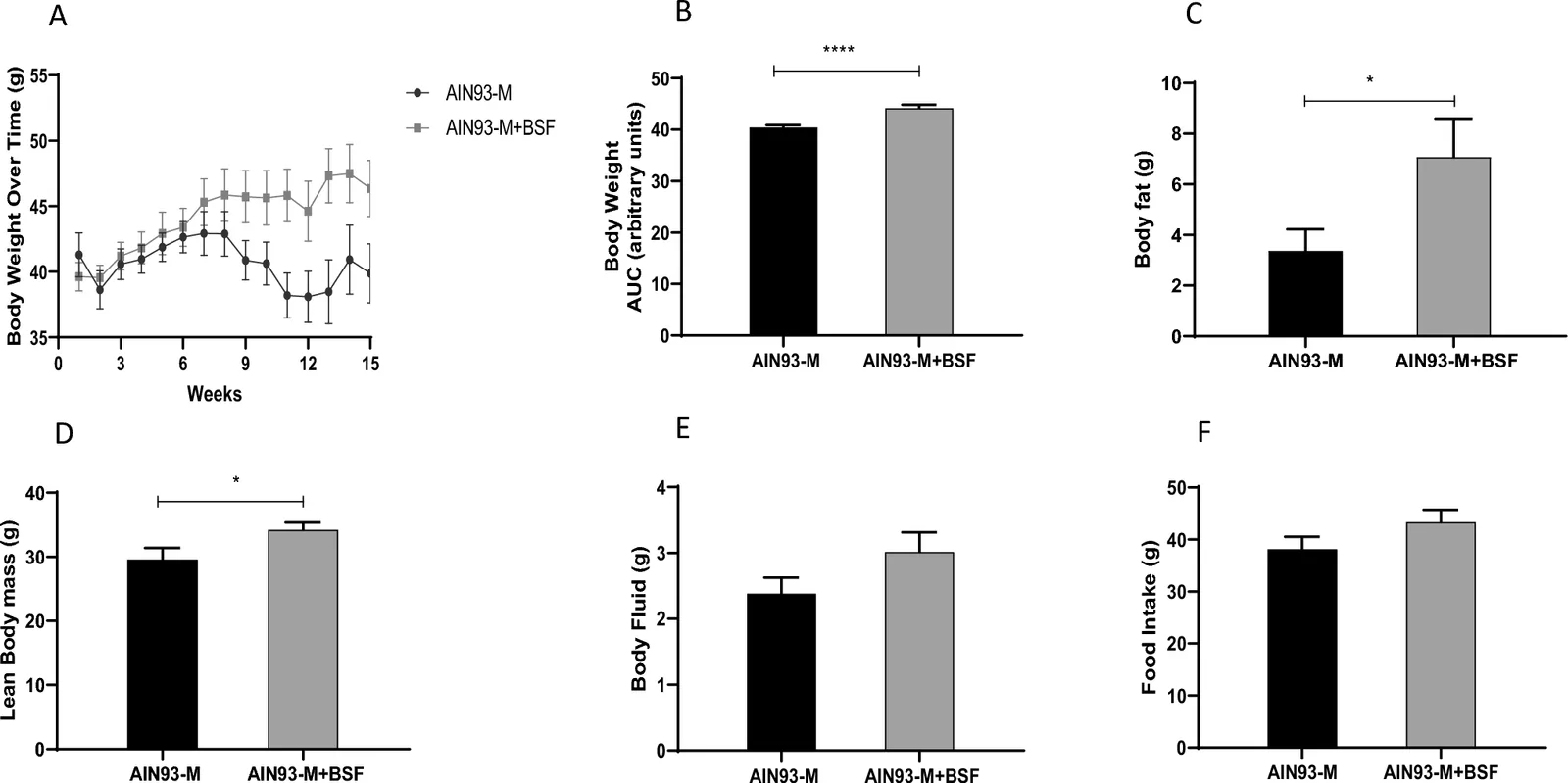
Dietary supplementation with *H. illucens* larvae meal increases body weight gain, adiposity, and lean mass without affecting food intake in mice. (**A**) Body weight progression during the experimental period. (**B**) Area under the curve (AUC) for body weight. (**C**) Fat mass. (**D**) Lean body mass. (**E**) Body fluid content. (**F**) Food intake. Data are expressed as mean ± SEM. Statistical significance was determined using Student’s *t*-test. *p < 0.05 and ****p < 0.0001 compared with the AIN93-M group (n=10)

Supplementation with *H. illucens* meal promoted a significant increase in body weight gain throughout the experimental period, evidenced by the larger area under the body weight curve (*p* < 0.0001) (Fig. 1A&B). Compared with controls, supplemented mice exhibited a 17.1% increase in body weight from baseline, reaching a final body weight of 46.36 g (p < 0.0001), whereas control mice showed a 3.5% reduction, with a final body weight of 39.87 g. Supplemented animals showed greater fat mass (*p* = 0.05) (Fig. 1C) and greater lean mass (*p* = 0.04) (Fig. 1D) compared to the control group. No significant differences were observed in body water (*p* = 0,12) content or total food intake ( p = 0,14) between the groups (Fig. 1E&F).

### Glycemic homeostasis

**Fig. 2:**
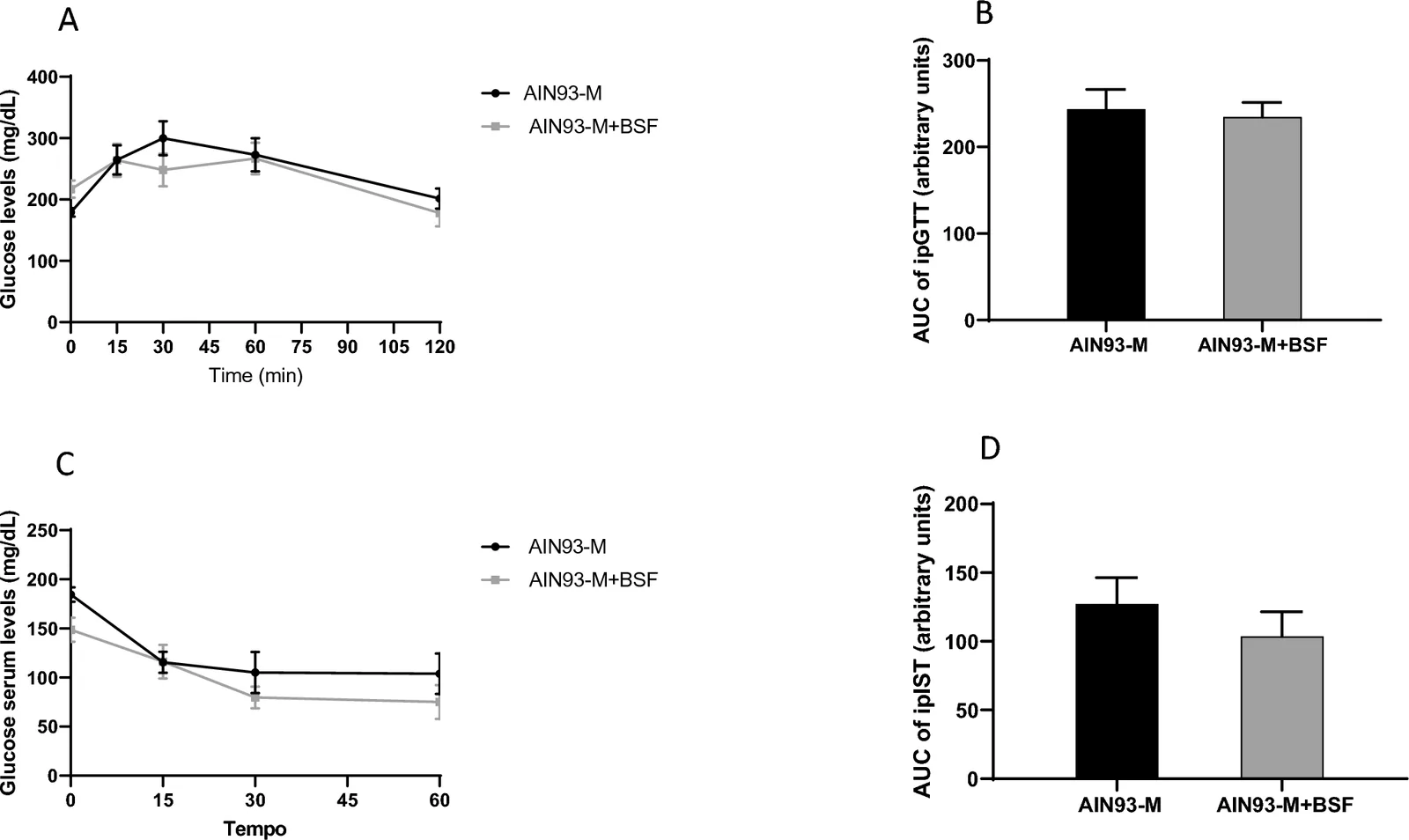
Dietary supplementation with *H. illucens* larvae meal does not alter glucose tolerance or insulin sensitivity in mice maintained under basal conditions. (**A**) Oral glucose tolerance test (GTT). (**B**) Area under the curve (AUC) of the GTT. (**C**) Insulin tolerance test (IST). (**D**) Area under the curve (AUC) of the IST. Blood glucose concentrations were monitored at the indicated time points following glucose or insulin administration. Data are expressed as mean ± SEM. No significant differences were observed between the AIN93-M and AIN93-M+BSF groups (n=10)

Glucose tolerance tests (GTT) and insulin sensitivity tests (IST) showed no significant differences between the experimental groups, as observed by the glycemic curves over time (Fig. 2A&C). Consistently, the area under the curve (AUC) values ​​remained similar between the groups for both tests, GTT (*p* = 0.76) and IST (*p* = 0.69) (Fig. 2B&D), indicating that supplementation with *H. illucens* flour did not promote changes in glucose tolerance or insulin sensitivity under basal conditions.

### Effects of H. illucens larval meal on serum biochemical parameters

Serum biochemical analysis revealed no significant differences between the Control (AIN-93 M) group and the *H. illucens* larval meal-supplemented group (AIN-93 M + BSF) for total cholesterol (*p* = 0.14), triglycerides (*p* = 0.07), aspartate aminotransferase (AST; *p* = 0.39), or alanine aminotransferase (ALT; *p* = 0.09). These findings indicate that dietary supplementation with *H. illucens* larval meal did not significantly alter serum lipid profile or liver function under the experimental conditions.

### H. illucens Supplementation modulates hepatic lipid homeostasis

**Fig. 3:**
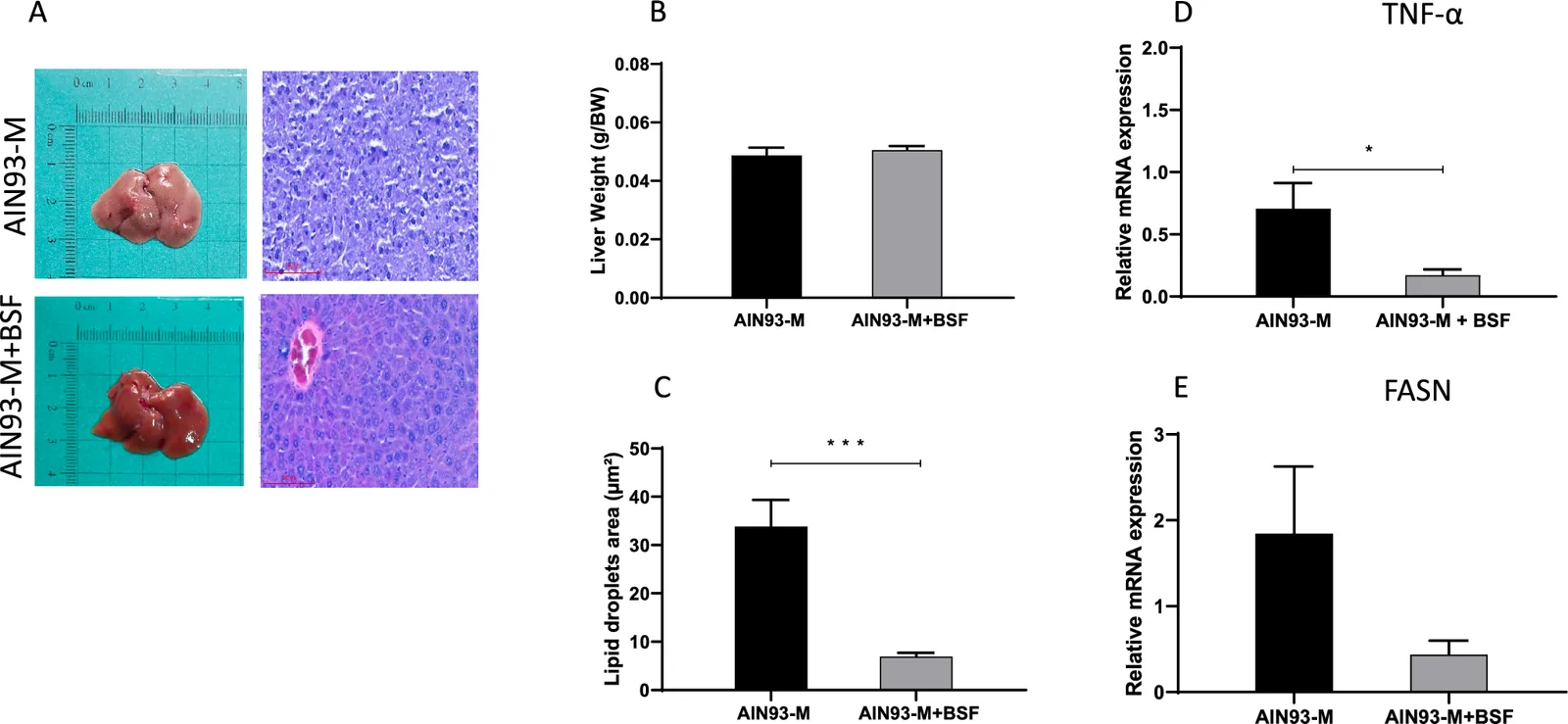
- Effects of *H. illucens* larvae meal supplementation on liver morphology, hepatic lipid accumulation, and expression of metabolic and inflammatory markers in mice. (**A**) Representative macroscopic images and histological sections (H&E staining, 400× magnification) of liver tissue from the control group (AIN93-M) and the group supplemented with *H. illucens* larvae meal (AIN93-M+BSF). (**B**) Relative liver weight (g/BW). (**C**) Quantification of hepatic lipid droplet area (µm^2^). (**D**) Relative hepatic mRNA expression of TNF-α. (**E**) Relative hepatic mRNA expression of FASN. Data are expressed as mean ± SEM. Statistical significance was determined using Student’s *t*-test. *p < 0.05 and ***p < 0.001 compared with the AIN93-M group (n=10)

Supplementation with *H. illucens* meal did not alter the relative liver weight between the experimental groups (Fig. 3B). However, histological analysis revealed a significant reduction in the area occupied by hepatic lipid droplets in supplemented animals (*p* = 0.009) (Fig. 3A&C), indicating less lipid accumulation in the liver tissue. At the molecular level, a significant reduction in hepatic TNF-α expression was observed (*p* = 0.01) (Fig. 3D), suggesting attenuation of inflammatory signaling. Additionally, FASN expression showed a tendency to decrease in supplemented animals ( *p* = 0,16), although without a statistically significant difference (Fig. 3E).

## Discussion

The *H. illucens* larval meal composition observed in the present study confirms its high nutritional value, as reflected by its elevated energy density and the predominance of lipids and proteins, which constitute the major nutritional fractions of the insect flour. The nutritional profile is consistent with previous studies that describe *H. illucens* as a nutrient-dense ingredient with considerable potential for both animal feed and human food applications [38, 39]. Moreover, the low moisture content observed is an important quality attribute, as it enhances microbiological stability and extends shelf life by reducing the risk of deterioration during storage [40]. The ash content also indicates that the flour may be a valuable source of essential minerals [41], further enhancing its nutritional value. Taken together, these characteristics reinforce the potential of *H. illucens* meal as a sustainable alternative ingredient capable of supplying high-quality nutrients while supporting the development of more environmentally sustainable food and feed systems.

The present study showed that dietary supplementation with *H. illucens* larval meal induced significant changes in body composition and hepatic metabolism in mice maintained under basal conditions. Supplemented animals displayed greater body weight gain, accompanied by increases in both lean and adipose mass, without changes in food intake. Given that the experimental diets were formulated to provide similar energy content, these findings suggest that the nutritional characteristics of *H. illucens* meal, including its high-quality protein fraction and lipid composition, may have contributed to the observed changes in tissue accretion. The protein fraction of *H. illucens* larval is characterised by a favorable amino acid profile and high biological value, which may support tissue development and metabolic maintenance under adequate nutritional conditions [42]. Similar increases in body weight gain and growth performance have been reported in other studies following dietary inclusion of *H. illucens* larval meal, supporting its potential as a source of bioavailable nutrients for tissue deposition and physiological development [43]. In this context, high-quality protein intake may contribute to the preservation and accretion of lean mass by supplying essential amino acids required for muscle protein synthesis, whereas increased availability of dietary fatty acids may favour energy storage and adipose tissue deposition, particularly under basal physiological conditions with limited energy expenditure [44, 45].

Despite the greater body weight and fat mass, glucose tolerance, insulin sensitivity, serum lipid concentrations, and liver enzyme activities remained unchanged, indicating that supplementation did not impair systemic metabolic homeostasis under physiological conditions. These findings are consistent with previous studies demonstrating that *H. illucens-*derived ingredients do not adversely affect metabolic or biochemical parameters while modulating hepatic lipid metabolism [46].

Dietary supplementation with *H. illucens* larval meal significantly reduced hepatic lipid droplet accumulation, whereas relative liver weight remained unchanged. These findings suggest that supplementation modulated hepatic lipid storage without affecting liver mass. Similar effects have been reported in studies evaluating *H. illucens*-derived lipids, which associated lower hepatic lipid accumulation with the predominance of medium-chain fatty acids, particularly lauric acid ( [18, 47]. Although these studies were performed under conditions of metabolic challenge, the present findings indicate that *H. illucens* may also influence hepatic lipid homeostasis under physiological conditions.

The reduction in hepatic lipid droplets was accompanied by lower hepatic TNF-α expression, suggesting attenuation of basal inflammatory signalling. Although FASN expression showed only a non-significant downward trend, these findings indicate that the lower hepatic lipid accumulation was not necessarily mediated by reduced de novo lipogenesis alone [48]. Similar molecular responses have been reported in experimental studies, in which reduced hepatic TNF-α expression was accompanied by lower hepatic lipid accumulation despite limited changes in lipogenic pathways, suggesting that modulation of inflammatory signalling also contributes to the maintenance of hepatic lipid homeostasis [49]. Collectively, the present results demonstrate that *H. illucens* larval meal promoted greater body-weight gain without compromising metabolic homeostasis, while reducing hepatic lipid accumulation and inflammatory signalling, supporting its potential as a functional ingredient for modulating hepatic lipid metabolism in healthy animals.

## Conclusion

Dietary supplementation with *H. illucens* larval meal increased body weight gain and lean and fat mass without altering glucose homeostasis or liver function in healthy mice. Notably, supplementation reduced hepatic lipid droplet accumulation and TNF-α expression, indicating modulation of hepatic lipid metabolism and inflammatory signaling under basal conditions. These findings highlight the potential of *H. illucens* larval meal as a functional ingredient and provide a basis for further mechanistic studies.

## Supplementary Information

Below is the link to the electronic supplementary material.Supplementary file1 Supplementary file2 

## Data Availability

No datasets were generated or analysed during the current study.
