# Supplementary material for: Dietary supplementation with black soldier fly (Hermetia illucens) larval meal modulates hepatic lipid homeostasis and reduces TNF-α expression in mice
Source: Mol Biol Rep. 2026 Aug 28;53(1):1478. doi: 10.1007/s11033-026-12657-2 (PMC13525021; doi:10.1007/s11033-026-12657-2)
Supplement: Supplementary file 1 [file 11033_2026_12657_MOESM1_ESM.docx]

**Supplementary Figure 1**. Sample size calculation performed using G*Power software (version 3.1.9.4) for the experimental design. The analysis was conducted to estimate the required number of animals per group based on the predefined statistical parameters.
