# Supplementary material for: Dietary supplementation with black soldier fly (Hermetia illucens) larval meal modulates hepatic lipid homeostasis and reduces TNF-α expression in mice
Source: Mol Biol Rep. 2026 Aug 28;53(1):1478. doi: 10.1007/s11033-026-12657-2 (PMC13525021; doi:10.1007/s11033-026-12657-2)
Supplement: Supplementary file 2 [file 11033_2026_12657_MOESM2_ESM.docx]

| **Run ID** | ***Assay Name** | **Test Name** | **RNA-IQ number** | **% Small RNA** | **%Large RNA** |
| --- | --- | --- | --- | --- | --- |
| 2026-03-15_222618 | G1 cab | Sample_#260715-225951 | 7.7 | 23% | 77% |
| 2026-03-15_222618 | G1 dor | Sample_#260715-224449 | 2.8 | 72% | 28% |
| 2026-03-15_222618 | G1 cau | Sample_#260715-223426 | 7.8 | 22% | 78% |
| 2026-03-15_222618 | G1 sm | Sample_#260715-223412 | 8.3 | 17% | 83% |
| 2026-03-15_222618 | G1 pde | Sample_#260715-223352 | 3.4 | 66% | 34% |
| 2026-03-15_222618 | G1 pdd | Sample_#260715-223339 | 8.9 | 11% | 89% |
| 2026-03-15_222618 | G1 ptd | Sample_#260715-223327 | 8.1 | 19% | 81% |
| 2026-03-15_222618 | G1 pte | Sample_#260715-223315 | 8 | 20% | 80% |
| 2026-03-15_222618 | G1 bar | Sample_#260715-223301 | 8.9 | 11% | 89% |
| 2026-03-15_222618 | G1 pes | Sample_#260715-223247 | 6.2 | 38% | 62% |
| 2026-03-15_222618 | G2 cab | Sample_#260715-223233 | 8 | 20% | 80% |
| 2026-03-15_222618 | G2 dor | Sample_#260715-223218 | 9 | 10% | 90% |
| 2026-03-15_222618 | G2 cau | Sample_#260715-223204 | 8.7 | 13% | 87% |
| 2026-03-15_222618 | G2 sm | Sample_#260715-223149 | 8.5 | 15% | 85% |
| 2026-03-15_222618 | G2 pdd | Sample_#260715-223133 | 7.1 | 29% | 71% |
| 2026-03-15_222618 | G2 pde | Sample_#260715-223115 | 6.2 | 38% | 62% |
| 2026-03-15_222618 | G2 ptd | Sample_#260715-222933 | 8.2 | 18% | 82% |
| 2026-03-15_222618 | G2 pte | Sample_#260715-222913 | 8.7 | 13% | 87% |
| 2026-03-15_222618 | G2 bar | Sample_#260715-222756 | 7.1 | 29% | 71% |
| 2026-03-15_222618 | G2 pes | Sample_#260715-222741 | 7.1 | 29% | 71% |

**Supplementary Table S1.** RNA integrity assessment of liver samples used for RT-qPCR analysis.

RNA integrity was evaluated using the Qubit™ RNA IQ Assay. **G1** corresponds to the **AIN-93M control group**, whereas **G2** corresponds to the **AIN-93M supplemented with 10% *H. illucens* larvae meal (BSF) group**. The abbreviations (**cab**, **dor**, **cau**, **sm**, **pde**, **pdd**, **ptd**, **pte**, **bar**, and **pes**) represent the identification codes assigned to individual animals (biological samples). RNA IQ values range from 1 to 10, with higher values indicating greater RNA integrity. The percentages of small and large RNA represent the proportion of degraded and intact RNA, respectively, as determined by the Qubit™ RNA IQ Assay.
